# Performability Evaluation of Load Balancing and Fail-over Strategies for Medical Information Systems with Edge/Fog Computing Using Stochastic Reward Nets

**DOI:** 10.3390/s21186253

**Published:** 2021-09-17

**Authors:** Tuan Anh Nguyen, Iure Fe, Carlos Brito, Vishnu Kumar Kaliappan, Eunmi Choi, Dugki Min, Jae Woo Lee, Francisco Airton Silva

**Affiliations:** 1 Konkuk Aerospace Design-Airworthiness Research Institute (KADA), Konkuk University, Seoul 05029, Korea; anhnt2407@konkuk.ac.kr (T.A.N.); vishnu@konkuk.ac.kr (V.K.K.); 2 Programa de Pós-Graduação em Ciência da Computação, Campus Universitário Ministro Petrônio Portella, Universidade Federal do Piauí (UFPI), Ininga, Teresina 64049-550, PI, Brazil; iuresf@gmail.com (I.F.); carlosvictor@ufpi.edu.br (C.B.); faps@ufpi.edu.br (F.A.S.); 3 School of Software, College of Computer Science, Kookmin University, Seoul 02707, Korea; emchoi@kookmin.ac.kr; 4 Department of Computer Science and Engineering, College of Engineering, Konkuk University, Seoul 05029, Korea; 5 Department of Aerospace Information Engineering, Konkuk University, Seoul 05029, Korea; jwlee@konkuk.ac.kr

**Keywords:** medical information system, edge/fog computing, performability evaluation, load balancing, fail-over mechanism, stochastic reward net, discrete-event simulation

## Abstract

The aggressive waves of ongoing world-wide virus pandemics urge us to conduct further studies on the performability of local computing infrastructures at hospitals/medical centers to provide a high level of assurance and trustworthiness of medical services and treatment to patients, and to help diminish the burden and chaos of medical management and operations. Previous studies contributed tremendous progress on the dependability quantification of existing computing paradigms (e.g., cloud, grid computing) at remote data centers, while a few works investigated the performance of provided medical services under the constraints of operational availability of devices and systems at local medical centers. Therefore, it is critical to rapidly develop appropriate models to quantify the operational metrics of medical services provided and sustained by medical information systems (MIS) even before practical implementation. In this paper, we propose a comprehensive performability SRN model of an edge/fog based MIS for the performability quantification of medical data transaction and services in local hospitals or medical centers. The model elaborates different failure modes of fog nodes and their VMs under the implementation of fail-over mechanisms. Sophisticated behaviors and dependencies between the performance and availability of data transactions are elaborated in a comprehensive manner when adopting three main load-balancing techniques including: (i) probability-based, (ii) random-based and (iii) shortest queue-based approaches for medical data distribution from edge to fog layers along with/without fail-over mechanisms in the cases of component failures at two levels of fog nodes and fog virtual machines (VMs). Different performability metrics of interest are analyzed including (i) recover token rate, (ii) mean response time, (iii) drop probability, (iv) throughput, (v) queue utilization of network devices and fog nodes to assimilate the impact of load-balancing techniques and fail-over mechanisms. Discrete-event simulation results highlight the effectiveness of the combination of these for enhancing the performability of medical services provided by an MIS. Particularly, performability metrics of medical service continuity and quality are improved with fail-over mechanisms in the MIS while load balancing techniques help to enhance system performance metrics. The implementation of both load balancing techniques along with fail-over mechanisms provide better performability metrics compared to the separate cases. The harmony of the integrated strategies eventually provides the trustworthiness of medical services at a high level of performability. This study can help improve the design of MIS systems integrated with different load-balancing techniques and fail-over mechanisms to maintain continuous performance under the availability constraints of medical services with heavy computing workloads in local hospitals/medical centers, to combat with new waves of virus pandemics.

## 1. Introduction

### 1.1. Medical Information Systems (MIS)

The proliferation of aggressive virus pandemics (e.g., Covid-19) all over the world in past years has been causing a huge workload for medical professionals and medical information systems (MIS) in hospitals and medical centers. In MIS, a patient’s healthcare data are constantly collected, stored, and managed under electronic medical records (EMR) as the medical building blocks of the MIS for a doctor’s medical decision-making and treatment solutions, while a hospital’s medical activities and operational management are collaboratively and seamlessly assisted and conducted via a networked system [1]. The use of such an MIS is to improve the quality and effectiveness of healthcare services within and across hospitals and medical centers. The implementation of existing MIS often relies on an underlying sophisticated networked system associated with advanced computing paradigms (e.g., cloud/fog/edge computing) via dedicated communication links to remote data centers [2].

### 1.2. Computing Paradigms of MIS

Traditional MIS have mostly relied on mainframe computing systems as a form of centralized computing in which they play the role of a central data repository or hub in a medical data processing center placed in the IT department of a hospital [3]. However, such computing paradigms have inherent limitations such as scalability, maintenance, and operational cost not to mention the capabilities for business continuity and interoperability between MIS across different medical organizations [4]. Modern MIS in hospitals/medical centers have been adopting the Cloud-centric Internet of Things (CIoT), in which the MIS involves three main state-of-the-art technologies including embedded systems and middle-ware in body area networks (BAN) and cloud services (e.g., everything as a service (XaaS)) to provide scalable and flexible medical services and solutions to every single patient at any time during her/his treatment [5,6]. Nevertheless, the CIoT model turns out to limit itself due to intrinsic challenges including bandwidth, latency, uninterrupted, resource-constraints and security (BLURS) in healthcare services, especially in the context of nationwide or worldwide virus pandemics [7]. The emergence of fog and edge computing paradigms (FEC) in recent years offers a complete computing solution to the cloud in IoT-based MIS by removing the gap between the central cloud and the medical things at hospitals with five advantages including security, cognition, agility, latency, and efficiency (SCALE) to secure continuous and trustworthy medical services [8] In particular, fog computing is the computing paradigm for the local, shifting the capabilities of cloud computing from geo-distributed data centers to the extreme edge of the network where medical sensors and devices are located [9]. On the other hand, edge computing in harmony with fog computing plays the role of data gateways and a collector in charge of synthesizing and pre-processing medical data from heterogeneous medical sensors and devices and, subsequently, transmitting the formatted data to the fog layer for further processing and cross-platform collaboration. In this way, the cloud layer at remote data centers is involved in advanced processing tasks such as the analytics of terabytes of medical data, AI-based medical research and so forth. The involvement of FEC in local regions actually helps to reduce non-critical data traffic outward from a local medical organization while enhancing the performance and availability of critical processing tasks and local data traffic. Thus, FEC is a proper solution for computing in MIS, especially in the context of high-volume data transactions and a huge workload due to large-scale virus pandemics. A number of recent works highlighted the significance of adopting edge/fog computing paradigms in healthcare systems to combat the on-going worldwide Covid-19 pandemic and for future intelligent and reliable healthcare systems. The works [10,11,12] presented comprehensive challenges and future directions of the adoption of the intelligent edge computing paradigm to provide intelligent, real-time healthcare IoT services and solutions with satisfactory energy consumption and latency criteria. On the other hand, some recent works [13,14] discussed the important of fog computing paradigms for healthcare 4.0, in which the harmony in the integration of IoT devices with fog computing paradigms plays a vital role in many latency-sensitive healthcare services such as healthcare monitoring tasks to monitor oxygen and blood sugar levels, and so forth, and the prediction of diseases using machine learning techniques based on real-time collected symptoms. Kumar et al. [15] elaborated more promising healthcare services when the FEC continuum is adopted in the healthcare industry.

### 1.3. Performability of MIS in Practice

Without any exceptions, MIS are inevitably prone to partial failures and system crashes [16]. In the context of raging pandemics, the possibility of sustaining medical operations across a hospital/medical center to provide 24/7 medical services with high-availability and performance of its MIS is challenging but crucial, since any uncertain failure hitting the MIS with a large amount of constant data transactions can cause unexpected tangled medical operations and even a severe loss of human lives due to the lack of instant medical responses and reasonable decision-making. Practical reports have shown the capability of realistic MIS and operational incidences in medical centers. At the LDS Hospital in Salt Lake City, Utah, USA, a computerized hospital information system, called the Health Evaluation through Logical Programming (HELP), serves 17,000 logons per day with *99.85% up-time (13.14 h of downtime per year)* [17]. An investigation of electronic medical record downtime (EMRD) in a busy urban emergency department from May 2016 to December 2017 in [18] showed that a total of *58 h downtime* with *12 episodes of EMRD* occurred during the study period with approximately *5 h at unpredictable intervals*. Reported in [19], an unexpected downtime of the National Institutes of Health Clinical Center (NIH/CC) EHR system in US on 13 May 2010 eventually resulted in a sudden loss of access to clinical information for all patients with a potential effect on patient care and safety due to a hardware failure leading a severe corruption of primary and backup databases. These facts urge the assimilation of the nature of performance and availability related issues and their countermeasures in MIS through comprehensive modelling and assessment of system design even before real-world system construction.

### 1.4. Fail-Over and Load Balancing Strategies

In the context of medical systems under heavy workload and medical data traffic (as mentioned above), a loss or a latency of instant access to a patient’s health data constantly collected by IoT-based medical sensors/devices can cause severely incorrect decision-making in treatment. Therefore, the integration of fault-tolerance techniques in harmony with load-balancing strategies into the design of FEC-based MIS is of paramount importance to secure a high level of performability for medical information operations in hospitals/medical centers. The FEC-enabled MIS, in practice, is expected to be capable of failing over the data transactions in the cases of system/subsystem breakdown to secure service continuity, and of routing medical data onto healthy components in order to maintain high performance of medical services in extremely over-loaded and fragile circumstances. Lumpp et al. [4] introduced various fault-tolerant information system architectures to secure high availability (HA) and business continuity (BC) in virtualized systems. Grottke et al. [20] presented different fault-tolerant techniques for a variety of software faults in [20]. Wang et al. [21] proposed an adaptive and fault-tolerant data processing mechanism to secure the reliability of data transmission and processing speed. On the other hand, a number of other works performed different studies on load balancing strategies in existing computing paradigms. Shah and Prajapati [22] presented the reallocation and allocation of virtual machines (VMs) in cloud computing using load balancing algorithms for efficiency in [22]. Panda and Jana [23] proposed a probabilistic approach for load balanced task scheduling in cloud computing. The recent work in [24] proposed a dynamic resource allocation method in a fog computing environment. In the literature, the combination between load balancing and fail-over strategies was not investigated in a complete manner for fog/edge computing layers. However, in the context of medical environments, the harmony of such strategies is of paramount importance to secure the continuity and performance of medical services, especially when the workload increases due to large-scale aggressive virus pandemics. The main idea behind this is if load-balancing techniques help enhance the performance, the fail-over mechanisms provide an assurance of service continuity with the obtained performance.

### 1.5. Literature Review

Previous works in the literature have shown rapid progress in the evaluation of healthcare systems, mostly focusing on availability without an appropriate consideration of performance and strategic techniques to guarantee system performability for a high level of quality of services (QoS). Strielkina et al. [5] presented in [5,25] two of the first studies adopting queuing network models and Markov models respectively for the availability evaluation of IoT healthcare systems considering attacks on vulnerabilities. Santos et al. [26] proposed using a multi-objective optimization algorithm, NSGA-II, in combination with stochastic models, to optimize the system availability of a fog-cloud based IoT for healthcare. Pereira et al. [27] in their most recent work proposed comprehensive continuous time Markov chain (CTMC) models to investigate the availability of fog/edge computing nodes for drone-based facial recognition security systems. Only two recent works [2,28] presented comprehensive studies on the performance assessment of IoT-based healthcare systems with a consideration of the integration of cloud/fog/edge computing paradigms using M/M/c/K queuing network models for pure performance evaluation. Santos et al. [29] was one of the most recent studies on a performability evaluation of an IoT-based medical system.

### 1.6. Contributions

To the best of our knowledge, previous works in the literature have not shown comprehensive studies on the performance assessment using SRN under certain constraints of availability requirements (or in other words, performability evaluations) of healthcare systems at local hospitals/medical centers with the integration of fog/edge computing paradigms. Thus, this study can be distinguished as one of the studies at the very early stages in the research area on the performability assessment of healthcare systems regarding various evaluation metrics. This work extends the progress of studies on the performability assessment of MIS with the following contributions.

- Proposed a comprehensive **performability SRN model of an edge/fog based MIS** in local hospitals or medical centers. The model captures detailed medical data processing and transmission from local edge layer to local fog computing nodes.
- Elaborated **failure modes** of fog nodes and their hosted VMs along with **fail-over mechanisms** at fog node levels in the SRN system model to assimilate the impact and applicability of fail-over mechanisms to secure the continuity of medical data processing and transmission in MIS.
- Elaborated **three main load balancing techniques** to handle massive amounts of medical data transactions including (i) probability based, (ii) random based and (iii) shortest-queue based data distribution.
- Captured **sophisticated behaviors and dependencies** between performance and availability sub-models in a monolithic SRN system model using a set of guard functions, which enables the performability evaluation of the whole system at a high level of detail and comprehension.
- Performed various **discrete-event simulations and analyses** of the developed SRN system model using a set of reward functions to assimilate the system behaviors based on the evaluation of different performability metrics of interest including (i) recover token rate, (ii) mean response time, (iii) drop probability, (iv) throughput, (v) queue utilization of network devices and fog nodes.

### 1.7. Research Remarks

Through the analyses, we draw some findings as follows.

- The developed model is capable of capturing sophisticated behaviors and dependencies when assessing performability metrics with different load balancing and fail-over mechanisms in an MIS.
- The impact of fail-over mechanisms at fog nodes and VMs are clear to not allow request losses in a real-time medical response system. Particularly, the performability metrics related to medical service continuity and quality including mean response time (MRT), drop probability (DP) of requests, or queue utilization (QU) of network devices are apparently higher in the MIS with fail-over mechanisms.
- Load balancing techniques are revealed to be the key role in the improvement of system performance. The shortest queue technique outperforms in most of the cases, compared to the remaining LB techniques.
- Lastly, the implementation of both load balancing techniques and fail-over mechanisms brings about better performability metrics (e.g., MRT, DP, QU) compared to the cases without their combination. The case with the shortest queue load balancing technique and fail-over mechanisms outperforms in most of the analyses, compared to the other cases in particular.

The development of a comprehensive and monolithic SRN model along with the above analysis findings can help accelerate the design phases of practical medical information systems in hospitals and medical centers by providing proofs of concept for different system configurations even before its real-world construction.

### 1.8. Paper Structure

The paper is organized as follows. In Section 2, we present a discussion of selected works on performability assessments of healthcare systems. In Section 3, the overall system architecture of a typical MIS is detailed. The corresponding performability system model is proposed in Section 4. The analysis and simulation results are presented in Section 5. The paper is concluded in Section 6.

## 2. Related Works

The use of analytical models with performance optimization structures is not very common, as it requires deep knowledge of the modelling of complex mechanisms. Using Petri nets, there were a greater number of works with load balancing policies [30,31,32]; however, none of them have addressed the issue of the fail-over mechanism. The structure of fail-over with Petri nets has already been investigated in an isolated context of availability with machine live migration [33,34], without considering performance metrics. Besides, none of them have taken into account scheduling jobs. Therefore, the present work is the first to combine, in a single model, performability issues (performance + availability) based on the adoption of fail-over mechanisms and load balancing strategies. Panda and Jana [23] presented a load balanced task scheduling algorithm for cloud computing using a probabilistic approach. The work modelled the execution time of tasks on VMs as a probability load matrix. However, the work limited itself to the elaboration of stochastic behaviors in the integration of performance and availability modelling and analysis, which is our focus in this study. Entezari-Maleki et al. [35] proposed SRN models for the performance assessment of a grid computing environment in the presence of failure-repair of resources. The proposed models took into account three different scheduling schemes called random selection, non-preemptive priority, and pre-emptive priority for simultaneous task scheduling of tasks to processors. In an extended study, Entezari-Maleki et al. [36] presented an SRN model for performability evaluation of workflow scheduling in grids in which the failure-repair behavior of processors is considered along with a performance assessment of several metrics including blocking probability and service time of a resource. Nevertheless, the focus of this work was on grid computing while our focus is on FE computing paradigms for medical systems. We also elaborate more sophisticated behaviors related to both fail-over and load balancing techniques. Sun et al. [37] proposed a reliability-performance-energy correlation Markov model that captures random resource failures and recovery in a multiagent cloud system (MACS). The work suggested interesting research topics for the investigation of the correlation between different metrics of interest, which is also the main focus of our work. Furthermore, we incorporate sophisticated interdependencies between components at different levels using SRN, which is an advanced modelling methodology to reduce the complexity in Markov models. Tang and Xie [38] presented the most recent work on the availability and performance of a healthcare cloud-based IoT system using a Markov chain model. Nevertheless, the work did not elaborate on the sophisticated system architecture of the cloud while focusing on a body area network (BAN) and the failure-repair of its communication links to a cloud. Strielkina et al. [5] presented one of the very early studies on modelling a healthcare Cloud-centered IOT system using queuing theory. In an extended work, Strielkina et al. [25] investigated the availability of cloud-centered healthcare IoT systems in consideration of attacks on vulnerabilities. The authors developed Markov models to capture the streams of requests and attacks on vulnerabilities and a procedure of recovery in the healthcare IoT infrastructure. The papers exposed some limitations of the sophistication of data streams and the interdependency between the performance and availability of components. Li et al. [39] presented one of the specific works on the adoption of the Petri Net modelling methodology for a real-world cloud healthcare system in China. The work attempted to capture the state of patients and the relationship between medical processes and resources between a big tertiary hospital and small community hospitals through a telemedicine platform. Mahulea et al. [40] presented a modular Petri net modeling methodology for healthcare systems. The work highlighted the modulization in modelling healthcare services based on Petri net models. A recent work [41] presented the use of a colored Petri net to model and analyze medical resource allocation. One of the most recent works [42] adopted a timed colored Petri net modeling approach for the simulation and improvement of patients’ workflow in heart clinics during the Covid-19 pandemic. These works showed the significant adoption of Petri net variants in the modelling and analysis of healthcare services in real-world hospitals or medical centers. However, none of the works considered the data transactions throughout the computing systems in the medical centers. Santos et al. [26] presented a hierarchical model of a reliability block diagram (RBD) with a stochastic Petri Net (SPN) and a surrogate model to analyze and maximize the availability of an IoT infrastructure for healthcare associated with cloud-fog computing paradigms. Nevertheless, performance metrics were not considered in the work. In [43,44], Araujo et al. [43] presented a hierarchical modelling approach using RBD and SPN/CTMC models for the dependability quantification of an m-Health system. The studies considered various failure causes of mHealth systems, such as communications, battery discharge rates and timeouts, to see their impact on the performance of delivered services. Nguyen et al. [16] presented a comprehensive modelling and analysis framework for the dependability and security quantification of Internet of Medical Things (IoMT) associated with Cloud/Fog/Edge continuum. However, the work did not elaborate the involvement of performance evaluation for such medical infrastructures. The works [2,28] studied different pure performance metrics of an Internet of Health Things (IoHT) such as mean response time (MRT), resource utilization, service throughput and so forth. Nevertheless, the evaluation of pure performance metrics without an appropriate consideration of failure modes is optimistic and, in some cases, it is not sufficient, for instance in the case of massive data transactions as in MIS. Santos et al. [29], in the most recent work on the performability evaluation of IoT-based medical systems, proposed a simplified hierarchical model for availability and performance evaluation in a separate manner for cloud/fog layers. Our focus is on the fog/edge computing layers for data transaction in MIS where a massive amount of medical data and processes can be disrupted due to unbalanced load and discontinuity of back-end computing fog/edge infrastructure.

The summary and comparison of the above-mentioned related works on MIS and performability evaluation are presented in Table 1 to highlight the contributions of this study. We consider different factors featured for the performability evaluation of a computing system for medical purposes. Particularly, we investigated the features of the system in previous works related to computing infrastructures for medical purposes including: (i) system specification (or context of the study); (ii) mechanisms (if possible) and/or metrics for QoS enhancement; and (iii) methodology for modelling and evaluation. To distinguish the key contributions of this work, we investigated whether the previous works under consideration elaborated different metrics for performability assessment or only for performance/availability/reliability evaluation in an optimistic and separate manner. As observed, most of the previous works did not consider performability assessment of a computing infrastructure for medical purposes regarding a variety of metrics in a comprehensive manner. Our main focus in this study is to explore the missing parts in the literature.

## 3. A medical Information System Architecture

**Figure 1:**
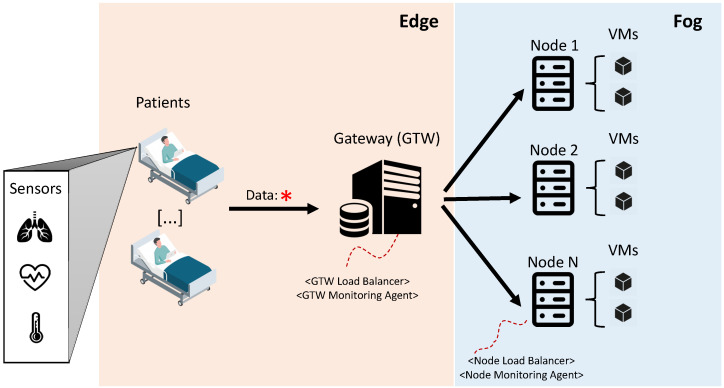
Overview of the scenario considered in the modelling phase.

This section presents an overview of the medical monitoring system architecture modeled through SRN. Figure 1 presents the architecture, divided into two layers: edge and fog. The edge encompasses data generation, and fog deals with the processing of such data. Edge monitoring tasks are operated upon a set of patients physically located within a hospital. Various health sensors are coupled to patients and generate vital data (e.g., temperature, pressure, heart rate), which are then transmitted to the fog through a gateway (GTW). The gateway, therefore, receives the data and relays it to the fog nodes. The gateway has two software modules: a module called GTW Load Balancer and a module called GTW Monitoring Agent. The GTW Load Balancer, as the name implies, makes the optimized distribution of data to the fog nodes obey a specific balancing strategy. The GTW Monitoring Agent constantly listens to how resources are being consumed at each node in the fog. Such monitoring is essential for the GTW Load Balancer to make appropriate decisions on how to distribute arriving data.

Upon reaching the fog, the data are transferred to a specific virtual machine (VM) by its respective host machine node. Therefore, the fog node also acts as a gateway since it has a monitoring module (*Node Monitoring Agent*). In the figure, fog nodes are represented by physical machines that host virtual machines. However, the proposed model abstracts the nature of such computing entities. The appraiser can define, for example, clusters comprised only of containers. However, for intuitive purposes, they are represented as physical nodes and VMs. The appraiser must feed the SRN model according to the characteristics of the adopted nodes. The appraiser is able to define a specific number of fog nodes in the modelling. Furthermore, both physical nodes and VMs can fail. In this case, specific data forwarding policies are adopted for both nodes and VMs.

**Figure 2:**
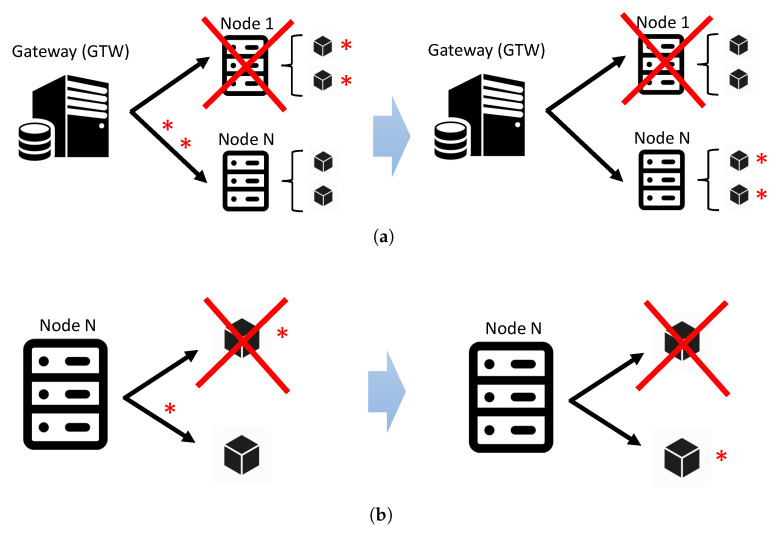
Data forwarding policies in the case of fog node and VM failures. (**a**) Scenario A: Node fail or all VMs fail; (**b**) Scenario B: One VM fails.

Figure 2 visualizes two scenarios in which the fog reacts to uncertain failures at either fog node or VM levels. As mentioned earlier, the gateway is responsible for monitoring physical nodes. Thus, if the GTW Monitoring Agent identifies a node in a failure mode (or all VMs in a node in failure mode), the GTW Load Balancer is triggered. All data previously sent to that nodeare forwarded immediately to another healthy node once again, as depicted in Figure 2a. The second failure scenario (Figure 2b) occurs when a single VM fails. In this case, the physical node of the fog is responsible for forwarding the data of the failed VM to another available VM, often obeying a sophisticated routing strategy.

## 4. A Performability SRN Model

**Figure 3:**
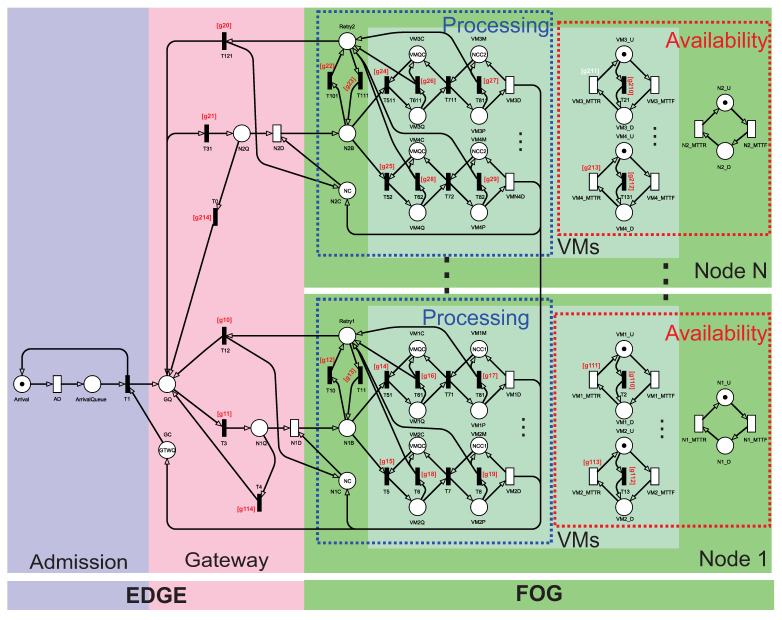
Overall SRN system model.

Figure 3 presents the overall performability system model using SRN. The model is divided into three sequential layers. For better visualization, different colors are used for each layer. The data (represented by tokens) are generated at the Admission layer, then the data are transmitted to the gateway, and finally, they are processed at the fog layer. Both physical machines (nodes) and respective virtual machines are represented in the model. There is a separation into sub-models that represent the processing and availability of nodes and VMs. In general, at the Admission, data are generated following an exponential distribution. When they arrive at the gateway, these data are redirected to one of the nodes. Such re-transmission follows a specific load balancing algorithm. Next, each sub-module is better detailed from the cut-outs of the system model. The model has several guard expressions used to control the token’s flow. All guard expressions follow the pattern “[gNK]”, where N means the node number (1 for fog node in the downside or 2 for the node in the upside). The letter K indicates a simple set of indexes for each node. The general guard expressions are summarized in Table 2. Table 3 presents the guard expressions related to the load balancing strategies. To ease the description of sophisticated behaviors captured in the models, we pick the two nodes in the fog layer and suppose that each node contains two VMs.

**Figure 4:**
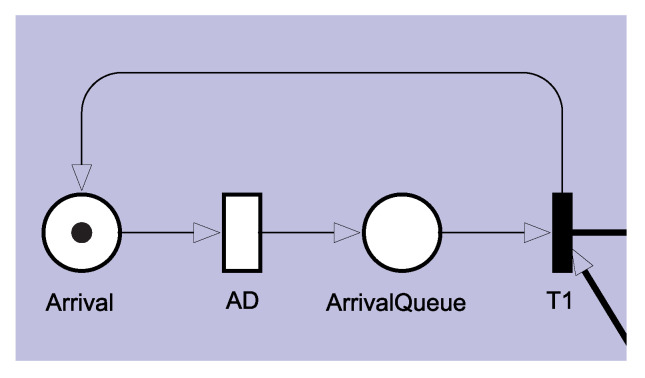
Admission submodule responsible for generating new requests following a specific probability distribution.

Figure 4 shows the Admission sub-model, composed of two places and two transitions. The Arrival place represents the arrival place for new requests. The timed transition AD represents the time between arrivals of each request. In the ArrivalQueue place, new requests are queued if the system is overloaded. If the system is not overloaded, the immediate transition T1 is triggered, and the token proceeds to enter the system, and a token is created at the Place Arrival to trigger a new request. The AD transition is configured following a single server policy, and therefore the arrival of requests occurs sequentially.

**Figure 5:**
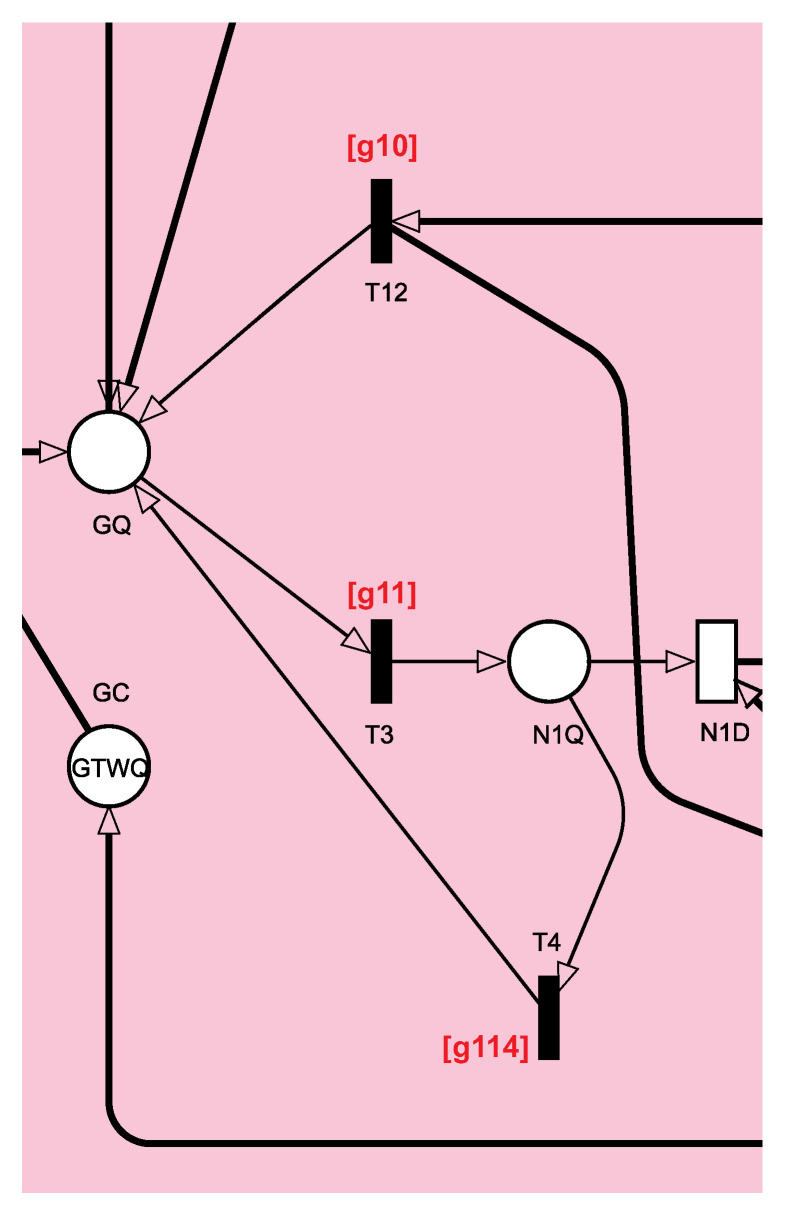
Gateway submodule responsible for transmitting requests to the final processing targets.

Figure 5 shows the cutout for the Gateway component. The gateway is responsible for choosing the node and storing the requests until the service is completed. Tokens in place GQ represent requests that have not yet defined their destination. For the token to arrive in GQ, the gateway must have available capacity, represented by the number of tokens in GC. After the consumption of tokens of the gateway’s capacity, that capacity will only be returned with the completion of the processing of the respective request. From the GQ location, tokens can go to one of the nodes when triggering any immediate transitions. For example, the T3 transition is triggered when node 1 is selected by the load balancing mechanism to proceed with processing. The guard expression of T3 is [g*11*] to guarantee the existence of tokens in the places VM1U or VM2U while there is a token in the place N1U at the same time. The guard expression [g*11*] means that the node is up and at least one of its VMs is up as well. The number of tokens in N1Q represents the requests that are in the gateway’s output buffer. From N1Q, the token can also return to the GQ place (T4 is triggered) if all VMs on the node are unavailable. The guard expression of T4 is [g*114*] to verify if there are zero tokens in both places VM1U and VM2U or no token in the place N1U. The T12 transition is triggered when the node or all VMs drop, and the fail-over mechanism requests the re-submission to the other node. The guard expression of T12 is [*g*10] as similar to [*g*114]. Finally, the N1D transition is triggered if there is capacity on the node and that node is available. N1D is an exponential transition that represents the time the data are sent over the network.

**Figure 6:**
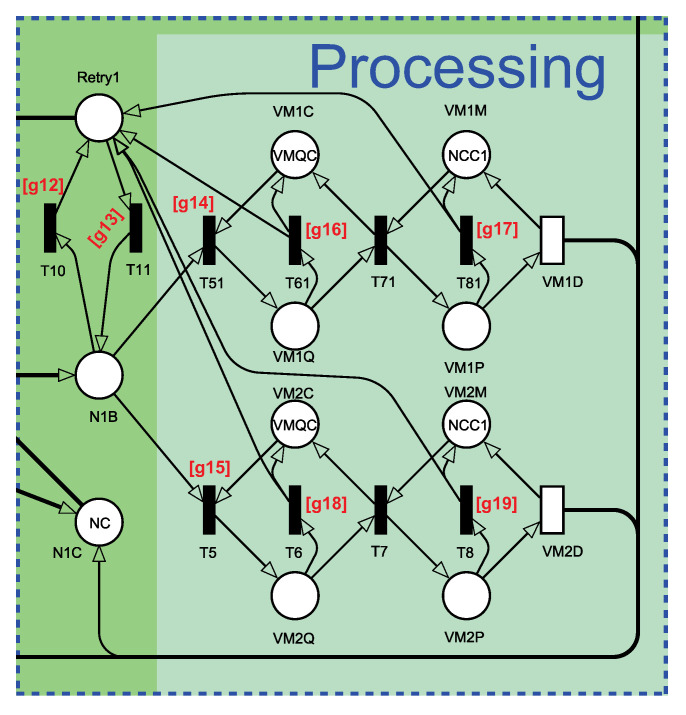
Processing submodule representing the virtual machines queues and processing capacities.

Figure 6 shows the processing sub-module. The number of tokens on N1C represents the capacity still available for processing on the node. When the token arrives at N1B, it must be chosen to process VM the request. Since the behavior of both VMs is similar, we will focus on explaining only the VM1 token flow. Considering that the token starts in place N1B and the decision made is to send a request to VM1, then transition T51 is triggered. The T51 transition is triggered if there is a queuing capability in the VM’s input buffer (place VM1C with marking VMQC). Therefore, tokens on VM1Q are waiting in line for the next processing step. The guard expression of T51 is [*g*14] to check if there is a token in the place VM1U. Such a guard expression means that VM1 is up. When T71 is triggered, the VM starts processing the request. The VM1D transition represents this processing time. When a VM goes down, all VMs return to the initial state, and their jobs must be re-sent to another VM (locally or on another node). The transitions T61 and T81 are triggered when the VM falls, also creating tokens in place of Retry1. Transitions T61 and T81 have the same guard expression [g*16*] = [g*17*] to monitor the cases in which the place VM1U has zero tokens. Considering that the token is still in N1B and there is no VM available on the node, the transition T10 is triggered, creating tokens on Retry1. The guard expression of T10 is [g*12*], which monitors the conditions if there is not a token in the places VM1U and VM2U. From Retry1, there are two possibilities. First, the request can be relayed to a VM internally on the same node. The second possibility is that the request is again available for forwarding through the gateway (GQ place) to another node with available VMs.

**Figure 7:**
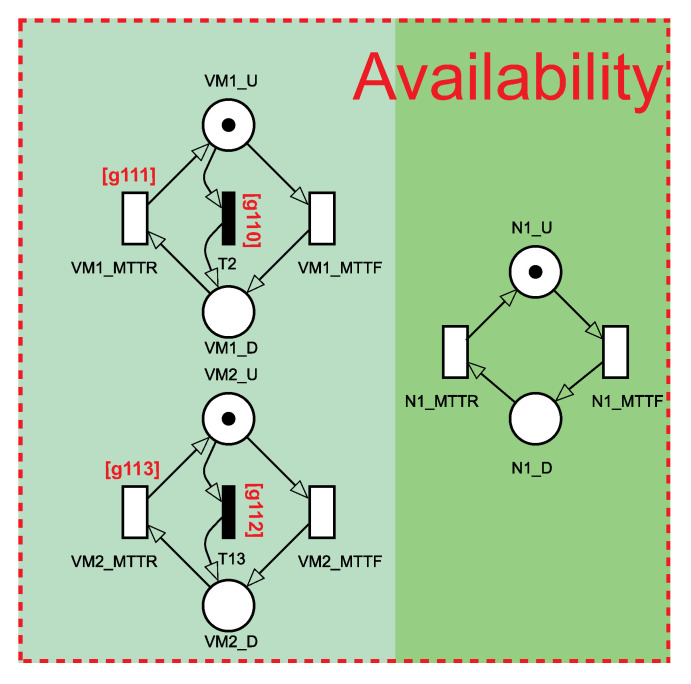
Availability sub-module encompassing availability aspects related to the fog node and respective virtual machines.

Figure 7 depicts the representation of availability to the node and respective VMs. On the left side, there are two building blocks for the two VMs, and on the right side, there is the building block to the node. For the three blocks, there are the traditional MTTF and MTTR transitions. Additionally, the VMs’ building blocks include immediate transitions to be triggered when the node fails. In this case, both VMs are turned off as a consequence. The guard expressions responsible for that are [g*110*] = [g*112*] to check if the place N1U has zero tokens. The recovery transitions (VM1_MTTR and VM2_MTTR ) present the contrary guard expressions: [g*111*] = [g*113*] to monitor if there is a token in the place N1U. All the fail-over mechanisms and load balancing strategies take this sub-module into account.

### Metrics

Table 4 presents the metrics used in the model. The mean response time (**MRT**) can be obtained by following Little’s law [45]. One of the most used theorems in queuing theory is Little’s law, which allows us to relate the mean number of jobs in any system with the mean time spent in the system as follows:mean number of jobs in the system equals the arrival rate timed mean response time. This relationship applies to all systems or parts of systems in which the number of jobs entering the system is equal to those completing service. Little’s law, which was first proven by Little (1961), is based on a black-box view of the system. The law applies as long as the number of jobs entering the system is equal to those completing service so that no new jobs are created in the system, and no jobs are lost forever inside the system. Even in systems where some jobs are lost due to finite buffers, the law can be applied to the part of the system consisting of the waiting and servingcomponents, because once a job finds a waiting position (buffer), it is notdropped. The arrival rate, in this case, should be adjusted to exclude jobs lost before finding a buffer. In other words, the effective arrival rate of jobs entering the system should be used. Therefore, by Little’s law, we can compute the mean response time as follows:

(1) $$MRT=(\frac{RequestsInProgress\times ArrivalDelay}{1-DropProbability}).$$

On the other hand, the RequestsInProgress is calculated by summing up all the expected number of tokens in each request-in-progress place. The arrival delay is the inverse of the arrival rate:

(2) $$ArrivalDelay=\frac{1}{ARR}.$$

It is noteworthy that Little’s Law requires the consideration of an effective arrival rate without considering dropped requests. Thus, as recommended by the author Jain (1990), we discount the drop probability by dividing the MRT by 1−P(#GC=0).

The throughput (**Tp**) represents how many requests per unit time one application can execute. This metric is obtained based on [46]. Tp is obtained by computing the expected value of tokens at a place, multiplied by the inverse of the subsequent transition delay. As in the system model, we present four VMs. We have the total throughput being the aggregation of the four throughput values. The drop probability (**DP**), as aforementioned, is the probability of discarding requests, calculated as the probability of not having available resources at the entering point of the system (the gateway).

Therefore, the recovered token rate (**RTR**) is calculated by adding the recovered token rate of all nodes. The **RTR** of one node (e.g., RTR_TotalNode01) is given by adding the token rate of all respective VMs and the node itself. The token rate of the VM (e.g., RTR_VM1) is given by multiplying the VM fail rate by the tokens that were executing at that moment. The VM fail rate (e.g., FailRateVM1) is obtained by multiplying the expected number of available VMs by the inverse of the MTTF. TokensNumberInProcess adds the tokens waiting in the queue and the tokens in the processing stage. The gateway queue utilization (**GQU**) is calculated by the number of tokens in the processing stage divided by the total gateway capacity. The node queue utilization (**NQU**) is the mean utilization of the respective nodes’ VMs.

## 5. Simulation Results

**Figure 8:**
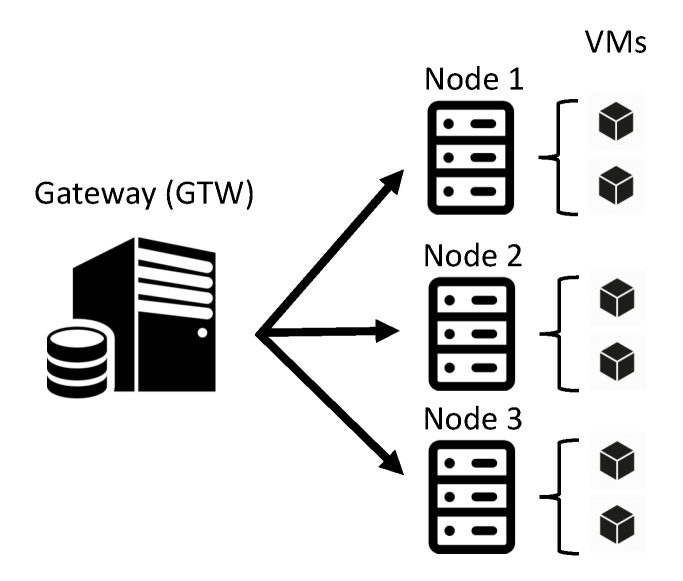
Case-study in simulation.

This section presents a set of simulation results on the proposed model. The models and simulations were performed using the Mercury tool [47] associated with discrete-event simulation engines. An MIS system with three fog nodes with two VMs in each node is our target system for the simulation as illustrated in Figure 8. The model with three nodes can better show the benefits and drawbacks of distinct optimization strategies.

Five metrics were adopted to achieve a complete understanding of the system. The adopted metrics are drop probability, recovered token rate, mean response time, throughput, and utilization (gateway and VMs). A comparison is performed based on each metric according to two aspects: load balancing strategies (three options) and use with and without a fail-over mechanism. Table 5 summarizes the model’s configuration combinations. This combination aims to compare the impact of using fail-over mechanisms with different classic load balancing strategies. Table 6 presents all the parameters used to feed the model before executing the simulations. Both time and resource capacities are detailed. The parameters were extracted from the literature ([26,48,49,50]).

**Figure 9:**
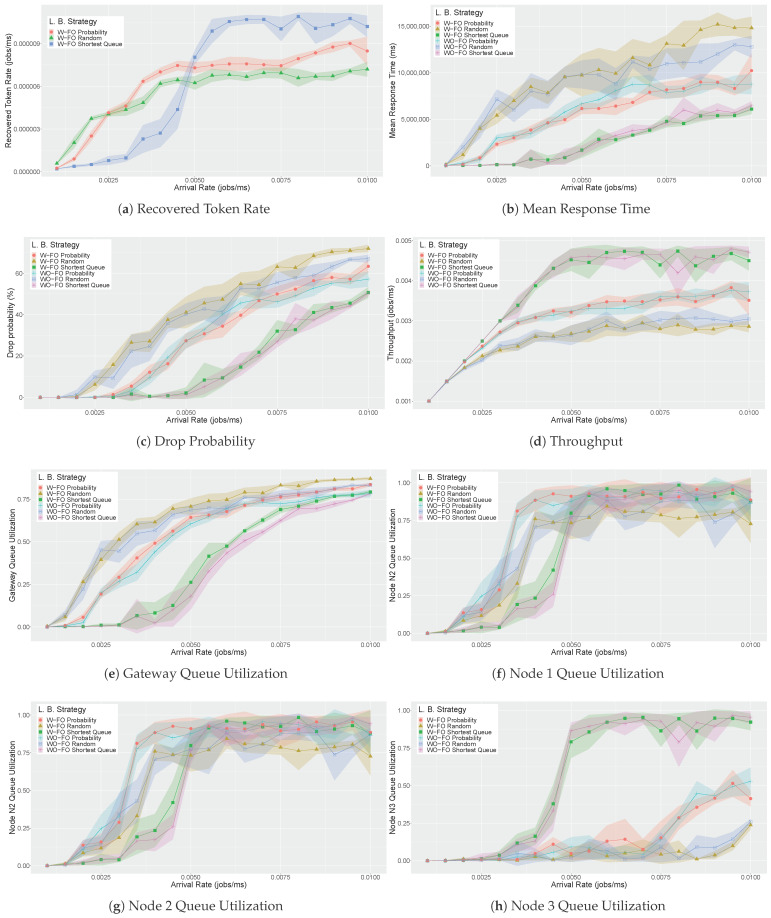
Simulation results for case-studies with/without load balancing strategies and fail-over mechanisms.

Figure 9 presents eight results, considering five metrics as previously mentioned. A variation on the arrival rate (ARR) was performed between 0 and 0.01 ms. A confidence interval accompanies all resulting lines. All metrics have a strong relationship with each other. The first point to note is comparing the adoption or not-adoption of the fail-over mechanisms (W−FO & WO−FO). Figure 9a shows the recovered token’s rate (RTR) with the fail-over mechanism activated. Without the fail-over mechanism, all these requests would be lost, so the graph shows only the results with the mechanism. The fail-over mechanism brings a small price to pay. In most points, there is a clear overlap of the results. However, there are points of non-overlapping in the metrics MRT, drop probability, and gateway queue utilization. In these cases, using the fail-over mechanism degrades performance somewhat to have a greater MRT, drop probability, and gateway queue utilization than when the fail-over mechanism is not used. This difference is observed mainly with the random load balancing strategy (ARR>0.0075 jobs/ms). In particular, the gateway queue utilization presents a higher number of different results between WO−FO and W−FO. Although such results are at first negative, two aspects must be observed. First, the difference with request loss happens only with very high arrival rates (ARR>0.0075 jobs/ms). Second, such a difference is a small price paid to not lose requests by re-sending them to be reprocessed when the system fails.

In Figure 9a, there is a significant difference between the behaviors of the three load balancing strategies. The probability based strategy recovers more requests than the Random based strategy because the Probability based strategy is more optimized than the Random based strategy. However, from ARR=0.005 jobs/ms, the number of requests recovered by the Shortest Queue strategy is even greater than the others. Probability causes more requests to be forwarded to more capable nodes, so more requests remain in the system and are not lost right at the gateway’s entrance. Even though it exposes flexibility, the Probability strategy is not adaptable to the arrival rate. The Shortest Queue, in turn, allows requests to be forwarded to less busy machines regardless of the arrival rate. The other metrics can corroborate this fact. Random’s MRT (Figure 9b) is greater than Probability, which is greater than Shortest Queue, regardless of arrival rate. The same justification applies to the drop probability (Figure 9c), where Random loses more requests than Probability, which loses more requests than Shortest Queue. The throughput (Figure 9d) of Shortest Queue is greater than Probability, which is greater than Random. The use of the gateway (Figure 9e) with Shortest Queue is less than the others because the nodes have lower mean utilization than the other two cases.

The analysis of the utilization of the three nodes (Figure 9f,g,h shows an interesting result regarding the load balancing strategies. First, it is worth remembering that the capacity of nodes 1, 2, and 3 are 8, 12, and 16 processors, respectively. For the Random and Probability strategies, node 1 reaches very high usage peaks earlier than node 2, while in node 3, this peak is not even reached. Random and Probability do not adapt to the increase in the arrival rate. It is worth mentioning that in node 2, Probability reaches 100% of utilization, but Random reaches only 75% because Random is not performative. The Shortest Queue, in turn, uses all the resources of node 3 as soon as nodes 1 and 2 no longer have available capacity. For this reason, the throughput (Figure 9d) of Shortest Queue is always better. Even with very low utilization with Random and Probability at Node 3, there is still a greater utilization of Probability than Random from the point ARR=0.0075 jobs/ms. Such an optimized capacity utilization of Shortest Queue explains why the Recovered Token Rate (Figure 9a) of this strategy is much better than the other when the system is highly busy (ARR>0.005 jobs/ms).

In summary, the simulations showed that the model is capable of calculating performance metrics with different load balancing techniques and fail-over mechanisms. As previously mentioned, the results must be observed broadly, encompassing all metrics. The more adaptable load balancing strategies to the workload tend to perform better and use resources better. The Shortest Queue strategy was superior in terms of RTR, mainly for higher arrival rates. The Shortest Queue obtained lower levels of drop probability at all points of arrival rate, as well as this strategy allowing greater throughput. Only three load balancing strategies were used, but other strategies can be applied to the model simply by configuring guard expressions and weight conditions. The contribution of the fail-over mechanism is clear because, in a real-time medical response system, request losses are not allowed. For example, observing the RTR (Figure 9a) for ARR=0.01 jobs/ms, we have that the fail-over mechanism with Shortest Queue avoids losing more than 40 requests per hour in the case of VM failure.

In the simulations, only the fail-over mechanism presence, the load balancing strategies, and arrival rates were varied. However, it is important to note that the appraiser can calibrate many other parameters in the model. As Table 6 shows, there are several parameters regarding service times and resource capacities. Of course, some parameters have a greater impact on metrics than others. In this case, it should be valuable to carry out a sensitivity analysis [51,52,53] to find out where the appraiser should invest in optimizing the system’s behavior. Another topic is the abstraction that the model allows obtaining. The computational nodes that the model refers to in principle refer to physical machines located in a local structure called a fog. However, such nodes can be located at a greater distance in a cloud structure since there are transitions regarding the transfer of data from the sensors to the processing nodes. The appraiser can, instead of exploring the use of virtual machines, also consider containers. To apply such an approach, the appraiser should only perform initial experiments with containers to obtain average response times and feed the model. Something that was not explored in the present simulations was the change of parameters related to availability (MTTF/MTTR) or extending the model by exploring the application of redundancies. All of these actions can impact the entire set ofstudied metrics.

## 6. Conclusions

In this paper, we proposed a comprehensive SRN performability model for an edge/fog based MIS system. Three main load-balancing techniques, including (i) probability-based, (ii) random-based and (iii) shortest-queue based approaches with/without fail-over mechanisms at the levels of fog nodes and fog VMs, were adopted and elaborated in the system model to assimilate the impact of the strategies to obtain a high level of trustworthiness on performance continuity under harsh availability conditions. Performability metrics of interest were comprehensively analyzed including: (i) recover token rate; (ii) mean response time; (iii) drop probability; (iv) throughput; and (v) queue utilization of network devices and fog nodes. The simulation results indicated that: (i) the impact of fail-over mechanisms at fog nodes and VMs are clear to not allow request losses in a real-time medical response system; (ii) load balancing techniques are revealed to be the key role in the improvement of system performance; (iii) the implementation of both load balancing techniques and fail-over mechanisms brings about better performability metrics compared to the cases without their combination, specifically, the shortest-queue load-balancing technique with a fail-over mechanism outperforms in maintaining major performability metrics compared to other strategies; and (iv) the developed model is capable of capturing sophisticated behaviors and dependencies when assessing performability metrics with different load balancing and fail-over mechanisms in an MIS. This study, along with its findings, can assist system design and operational management in practical medical information systems to obtain better performance and availability of medical services in the chaotic circumstances of virus pandemics.

## Figures and Tables

**Table 1:** Selected Works in Comparison.

Works	System		Method.	Metrics					
	Spec.	QoS		Perf.	MRT	TP	DP	RTR	QU
[23]	Cloud computing systems	LB, LS	Pr	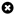	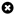	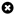	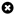	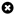	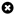
[37]	Cloud computing	reliability, performance, energy	QN, Markov chain	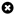	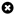	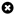	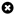	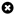	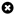
[38]	Healthcare IoT systems (Wireless body area network—WBAN)	availability performance improving (API) method (increasing probability of system full service)	Markov chain	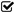	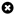	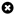	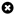	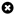	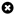
[5,25]	Healthcare IoT systems	Availability under attack vulnerabilities	Markov chain	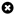	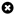	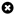	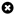	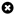	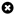
[26]	IoT for healthcare	Availability optimization combining stochastic models with optimization algorithms	RBD, PN, Surrogate models (extension of [29])	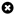	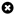	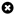	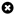	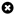	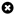
[43,44]	Mobile cloud computing for healthcare (mHealth)	Availability	RBD, PN	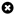	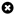	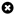	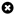	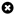	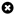
[16]	Cloud/Fog/Edge based IoT for healthcare monitoring	Reliability, availability, security under persistent software attacks	FT, CTMC	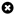	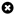	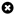	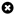	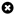	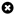
[28]	Fog-cloud IoT system for healthcare monitoring	Redundancy and scalability of fog/cloud for performance enhancement	QN	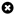	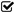	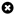	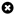	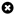	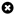
[29]	Edge/Fog/Cloud based e-Health IoT system	Availability, performance	RBD, PN	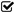	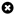	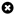	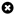	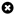	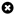
[35]	Grid computing environment (with failure-repair of resources)	LS (random selection, non-preemptive priority, and pre-emptive priority)	SRN	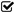	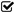	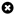	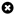	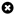	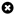
[36]	Grid computing	LS (genetic-based scheduling algorithm of programs)	SRN	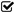	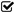	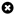	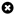	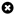	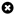
This work	Edge/Fog Medical Information System for healthcare	LB, Fail-over mechanisms	SRN	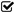	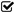	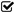	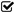	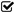	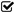

*Acronyms*—Spec.:System Specification; QoS: quality of service; LS: load/task scheduling; LB: load balancing; QN: queuing network; Pr: probabilistic methodology; RBD: reliability block diagram; FT: fault tree; CTMC: continuous-time Markov chain; SRN: stochastic reward net; Perf.: performability evaluation; MRT: mean response time; TP: throughput; DP: discard probability of jobs; RTR: recovered token rate; QU: queue utilization.

**Table 2:** Model Guard Expressions.

Transition	Index	Guard Expression	Module
T12	[g*10*]	((#VM1U=0) && (#VM2U=0))||(#N1U=0)	Gateway
T3	[g*11*]	((#VM1U=1)||(#VM2U=1)) && (#N1U=1)	Gateway
T10	[g*12*]	((#VM1U=0) && (#VM2U=0))	Node 1—Processing
T11	[g*13*]	((#VM1U=1)||(#VM2U=1)) && (#N1U=1)	Node 1—Processing
T51	[g*14*]	(#VM1U=1)	VMs—Processing
T5	[g*15*]	(#VM2U=1)	VMs—Processing
T61	[g*16*]	(#VM1U=0)	VMs—Processing
T81	[g*17*]	(#VM1U=0)	VMs—Processing
T6	[g*18*]	(#VM2U=0)	VMs—Processing
T8	[g*19*]	(#VM2U=0)	VMs—Processing
T2	[g*110*]	(#N1U=0)	VMs—Availability
VM1_MTTR	[g*111*]	(#N1U=1)	VMs—Availability
T13	[g*112*]	(#N1U=0)	VMs—Availability
VM2_MTTR	[g*113*]	(#N1U=1)	VMs—Availability
T4	[g*114*]	((#VM1U=0) && (#VM2U=0))||(#N1U=0)	Gateway
T121	[g*20*]	((#VM3U=0) && (#VM4U=0))||(#N2U=0)	Gateway
T101	[g*22*]	((#VM3U=0) && (#VM4U=0))	Node 2—Processing
T111	[g*23*]	((#VM3U=1)||(#VM4U=1)) && (#N2U=1)	Node 2—Processing
T511	[g*24*]	(#VM3U=1)	VMs—Processing
T52	[g*25*]	(#VM4U=1)	VMs—Processing
T611	[g*26*]	(#VM3U=0)	VMs—Processing
T811	[g*27*]	(#VM3U=0)	VMs—Processing
T62	[g*28*]	(#VM4U=0)	VMs—Processing
T82	[g*29*]	(#VM4U=0)	VMs—Processing
T21	[g*210*]	(#N2U=0)	VMs—Availability
VM3_MTTR	[g*211*]	(#N2U=1)	VMs—Availability
T131	[g*212*]	(#N2U=0)	VMs—Availability
VM4_MTTR	[g*213*]	(#N2U=1)	VMs—Availability
T0	[g*214*]	((#VM3U=0) && (#VM4U=0))||(#N2U=0)	Gateway

**Table 3:** Loading Balancing Strategies—Guard Expressions.

Load Balancing Strategy	Transition	Index	Node	Guard Expression
Shortest Queue	T3	[g*11*]	1	$\begin{array}{l} \mathrm{GENode}1=(\mathrm{Node}1\mathrm{UP}\;\&\&\;\mathrm{Node}1\mathrm{LessThenNode}2||\mathrm{Node}2\mathrm{Down})\\ \mathrm{Node}1\mathrm{UP}=((\#\mathrm{VM}1U\;=\;1)||(\#\mathrm{VM}2U\;=\;1)\;\&\&\;(\#N1U\;=\;1)\\ \mathrm{Node}1\mathrm{LessThenNode}2\;=\;((\#N1Q\;+\;\#N1B\;+\;\#\mathrm{VM}1Q\;+\;\#\mathrm{VM}2Q)\;\leq \\ \left(\#N2Q\;+\;\#N2B\;+\;\#\mathrm{VM}3Q\;+\;\#\mathrm{VM}4Q)\right)\\ \mathrm{Node}2\mathrm{Down}=(((\#\mathrm{VM}3U\;=\;0)\;\&\&\;(\#\mathrm{VM}4U\;=\;0) \end{array}$
	T31	[g*21*]	2	$\begin{array}{l} \mathrm{GENode}2=(\mathrm{Node}2\mathrm{UP}\;\&\&\;\mathrm{Node}2\mathrm{LessThenNode}1||\mathrm{Node}1\mathrm{Down})\\ \mathrm{Node}2\mathrm{UP}=((\#\mathrm{VM}3U\;=\;1)||(\#\mathrm{VM}4U\;=\;1)\;\&\&\;(\#N2U\;=\;1)\\ \mathrm{Node}2\mathrm{LessThenNode}1\;=\;((\#N2Q\;+\;\#N2B\;+\;\#\mathrm{VM}3Q\;+\;\#\mathrm{VM}4Q)\;\leq \\ \left(\#N1Q\;+\;\#N1B\;+\;\#\mathrm{VM}1Q\;+\;\#\mathrm{VM}2Q)\right)\\ \mathrm{Node}1\mathrm{Down}=(((\#\mathrm{VM}3U\;=\;0)\;\&\&\;(\#\mathrm{VM}4U\;=\;0) \end{array}$
Probability	T3	[g*11*]	1	No guard expression, only the probability percentage.
	T31	[g*21*]	2	No guard expression, only the probability percentage.
Random	T3	[g*11*]	1	GENode1=((#VM1U=1)OR(#VM2U=1))AND(#N1U=1)
	T31	[g*21*]	2	GENode2=((#VM3U=1)OR(#VM4U=1))AND(#N2U=1)

**Table 4:** Metrics used in the model.

Metric	Expression
MRT	$\mathrm{MRT}=(\frac{RequestsInProgress\times ArrivalDelay}{1---DropProbability})$ $\begin{array}{c} RequestsInProgress=\\ (\left(E\left\{\#GQ\right\}\right)+\left(E\left\{\#N1Q\right\}\right)+\left(E\left\{\#N1B\right\}\right)+\left(E\left\{\#VM1Q\right\}\right)+\\ \left(E\left\{\#VM1P\right\}\right)+\left(E\left\{\#VM2Q\right\}\right)+\left(E\left\{\#VM2P\right\}\right)+\left(E\left\{\#N2Q\right\}\right)+\\ \left(E\left\{\#N2B\right\}\right)+\left(E\left\{\#VM3Q\right\}\right)+\left(E\left\{\#VM3P\right\}\right)+\left(E\left\{\#VM4Q\right\}\right)+\\ \left(E\left\{\#VM4P\right\}\right)+\ldots +\left(E\left\{\#NNQ\right\}\right)+\left(E\left\{\#NNB\right\}\right)+\left(E\left\{\#VMMQ\right\}\right)+\\ \left(E\left\{\#VMMP\right\}\right)+\left(E\left\{\#VMM2Q\right\}\right)+\left(E\left\{\#VMM2P\right\}\right)) \end{array}$ $\begin{array}{c} \mathrm{ArrivalDelay} \end{array}=\frac{1}{ARR}$ DropProbability=P{(#GC=0)}
Through- put (Tp)	$\mathrm{Tp}=\sum_{0}^{N}(ExpectedNumberTokensToGoOut_{N}\times OutputRate_{N})$ Tp=((E{#VM1P})×(1/VM1D))+ $(\begin{array}{c} \left(E\{\#VM2P\}\right) \end{array}\times \begin{array}{c} \left(1/VM2D\right) \end{array})+$ $(\begin{array}{c} \left(E\{\#VM3P\}\right) \end{array}\times \begin{array}{c} \left(1/VM3D\right) \end{array})+$ $\left(\begin{array}{c} \left(E\{\#VM4P\}\right) \end{array}\times \begin{array}{c} \left(1/VM4D\right) \end{array}\right)$
Drop Probability (DP)	**DP** = P{(#GC=0)}
Recovered Token Rate (RTR)	RTR=RTR_TotalNode01+RTR_TotalNode02+RTR_TotalNodeN $\begin{array}{c} RTR\_TotalNode01 \end{array}=\left(\begin{array}{c} RTR\_VM1 \end{array}+RTR\_VM2\right)+\begin{array}{c} RTR\_Node01 \end{array}$ $\begin{array}{c} RTR\_VM1 \end{array}=\begin{array}{c} FailRateVM1 \end{array}\times \begin{array}{c} TokensNumberInProcessVM1 \end{array}$ $\begin{array}{c} FailRateVM1 \end{array}=\left(E\left\{\#VM1U\right\}\right)\times \left(1/VM1\_MTTF\right)$ $\begin{array}{c} TokensNumberInProcessVM1 \end{array}=\left(\left(E\left\{\#VM1Q\right\}\right)+\left(E\left\{\#VM1P\right\}\right)\right)$ $\begin{array}{c} RTR\_Node01 \end{array}=\begin{array}{c} FailRateNode01 \end{array}\times \begin{array}{c} TokensNumberInProcessNode01 \end{array}$ $\begin{array}{c} FailRateNode01 \end{array}=\left(E\left\{\#N1U\right\}\right)\times \left(1/NODEMTTF\right)$ $\begin{array}{c} TokensNumberInProcessNode01 \end{array}=\left(E\left\{\#N1B\right\}\right)$
Gateway Queue Utilization (GQU)	$\mathrm{GQU}=\frac{GTWQ-(E\{\#GC\})}{GTWQ}$
Node Queue Utilization (NQU)	$\mathrm{NQU}=\frac{\left((E\{\#VM1Q\})+(E\{\#VM2Q\})\right)}{\left(VMQC\times 2\right)}$

**Table 5:** Combinations of load balancing techniques and fail-over mechanisms to reduce request losses and enhance response time.

Feature	Name	Description
Load Balancing	Probability	Each node has an associated target probability. The assumed probabilities were: Node 1 = 25%, Node 2 = 35%, and Node 3 = 40%. The higher the node’s capacity is, the higher the probability percentage of forwarding requests to that node can obtain.
	Random	The node target is chosen randomly.
	Shortest Queue	The load balancing technique selects a fog node with the shortest queue capacity.
Fail-over	With Fail-over (W−FO)	The fail-over mechanism is adopted
	Without Fail-over (WO−FO)	The fail-over mechanism is not adopted.

**Table 6:** Default parameters of the SRN system model.

Parameter	Description	Time	Capacity
ARR	Arrival Rate	0.001–0.01 ms	n/a
N1D	Node 1 Transferring Time	1 s	n/a
VM1D & VM2D	Node 1 Service Time	30 s	n/a
N2D	Node 2 Transferring Time	1 s	n/a
VM3D & VM4D	Node 2 Service Time	20 s	n/a
N3D	Node 3 Transferring Time	1 s	n/a
VM5D & VM6D	Node 3 Service Time	10 s	n/a
NCC1	Node 1 Virtual Machine Capacity	n/a	8
NCC2	Node 2 Virtual Machine Capacity	n/a	12
NCC3	Node 3 Virtual Machine Capacity	n/a	16
NC	Node Capacity	n/a	1000
VMQC	Virtual Machine Queue Capacity	n/a	100
VMN_MTTF	Virtual Machine N Mean Time to Failure	1 day	n/a
VMN_MTTR	Virtual Machine N Mean Time to Repair	2 h	n/a
NN_MTTF	Node N Mean Time to Failure	7 days	n/a
NN_MTTR	Node N Mean Time to Repair	2 h	n/a
GTWQ	Gateway Queue Capacity	n/a	30,000
